# The Influence of Ouabain on Human Dendritic Cells Maturation

**DOI:** 10.1155/2014/494956

**Published:** 2014-12-28

**Authors:** C. R. Nascimento, R. C. Valente, J. Echevarria-Lima, C. F. L. Fontes, L. de Araujo-Martins, E. G. Araujo, V. M. Rumjanek

**Affiliations:** ^1^Instituto de Bioquímica Médica Leopoldo de Meis, Universidade Federal do Rio de Janeiro, 21 941-902 Rio de Janeiro, RJ, Brazil; ^2^Instituto de Biofísica Carlos Chagas Filho, Universidade Federal do Rio de Janeiro, 21 941-902 Rio de Janeiro, RJ, Brazil; ^3^Instituto de Microbiologia Paulo de Góes, Universidade Federal do Rio de Janeiro, 21 941-902 Rio de Janeiro, RJ, Brazil; ^4^Instituto de Biologia, Universidade Federal Fluminense, 24 230-340 Niterói, RJ, Brazil; ^5^Laboratorio de Imunologia Tumoral, Instituto de Bioquímica Médica Leopoldo de Meis, Centro de Ciencias da Saude, Bloco H02, Sala 003, Universidade Federal do Rio de Janeiro, 21 941-902 Rio de Janeiro, RJ, Brazil

## Abstract

Although known as a Na,K-ATPase inhibitor, several other cellular and systemic actions have been ascribed to the steroid Ouabain (Oua). Particularly in the immune system, our group showed that Ouabain acts on decreasing lymphocyte proliferation, synergizing with glucocorticoids in spontaneous thymocyte apoptosis, and also lessening CD14 expression and blocking CD16 upregulation on human monocytes. However, Ouabain effects on dendritic cells (DCs) were not explored so far. Considering the peculiar plasticity and the importance of DCs in immune responses, the aim of our study was to investigate DC maturation under Ouabain influence. To generate immature DCs, human monocytes were cultured with IL-4 and GM-CSF (5 days). To investigate Ouabain role on DC activation, DCs were stimulated with TNF-*α* for 48 h in the presence or absence of Ouabain. TNF-induced CD83 expression and IL-12 production were abolished in DCs incubated with 100 nM Ouabain, though DC functional capacity concerning lymphocyte activation remained unaltered. Nevertheless, TNF-*α*-induced antigen capture downregulation, another maturation marker, occurred even in the presence of Ouabain. Besides, Ouabain increased HLA-DR and CD86 expression, whereas CD80 expression was maintained. Collectively, our results suggest that DCs respond to Ouabain maturating into a distinct category, possibly contributing to the balance between immunity and tolerance.

## 1. Introduction

The appropriate functioning of the immune system relies, in several aspects, on the action of specialized antigen presenting cells, which promote the activation of effector cells and the enhancement and continuation of the immune response. Amongst them, dendritic cells (DCs) play the most important role in the uptake, process, and presentation of antigens to naïve CD4, CD8, and B cells, owing to their ability to induce such effect even with a relative small number and in the presence of low antigen levels [1].

DCs are derived from bone marrow progenitors and circulate in the blood as immature precursors prior to migration into peripheral tissues. They are originated from different hematopoietic lineages and are basically classified in two groups: the conventional and the plasmacytoid DCs. The first is often divided into subtypes, where subsets from different tissues present particular morphologies, phenotypes, location, cytokine production profiles, and functions [2]. These subtypes develop either as a result of the commitment of different cell precursors or by environmental cues that modify DC function, which in turn provide a larger range of possibilities to dictate the fate of lymphocytes. Specialized cell subtypes can induce an active response against several antigens [1] but tolerogenic properties have also been described for DCs [3, 4]. Thus, owing to the crucial role in the control of immunity, DCs are widely studied for clinical purposes that involve the function of T lymphocytes.

In this manner, the modulation of either the maturation and/or the activation and function of dendritic cells is of great importance and several studies have described some situations where DCs present such plasticity. For instance, DCs matured in the presence of vitamin D(3), IL-10, TGF-*β*, rapamycin, or dexamethasone were characterized as tolerogenic cells [4]. Additionally, conditions of low oxygen supply (hypoxia) may inhibit the expression of surface molecules closely related with the ability of differentiation and maturation of DCs, like MHC class II molecules, CD80, CD86, CD40, and CD1a [5]. The administration of sodium butyrate, a histone deacetylase compound that modifies chromatin organization, also deregulated DCs ability of upregulating CD1a protein during differentiation, a member of the CD1 family that mediate the presentation of lipid and glycolipid antigens, either from self or from pathogen origin, to CD1-restricted T lymphocytes [6]. Finally, DCs can also respond to several other endogenous stimuli, such as inflammatory mediators, neuropeptides, and hormones [7, 8]. The plasticity of DCs suggests that they might be also affected by drugs used in the treatment of chronic diseases.

The cardiotonic steroid Ouabain and other related glycosides have been in clinical use for many years for the management of congestive heart failure. Ouabain regulates myocyte contractility by inhibiting Na,K-ATPase activity, this being the basis of the therapeutic use of these kinds of drugs [9]. In addition to its role on myocyte contraction, several reports described the effect of Ouabain on immune cells and, as a result, Ouabain may be also considered an immunomodulatory substance [10–15] capable of modifying the inflammatory process [16–18]. Though classically described as a glycoside extracted from plants with a strong affinity for the Na,K-ATPase enzyme (Na,K-ATPase), studies have shown that mammals, including humans, possess an endogenous analog of plant-derived Ouabain in several tissues and also circulating in plasma [19, 20]. Ouabain levels are usually increased under acute stress and in pathophysiological conditions, like hypertension or even pregnancy [21, 22].

Although Na,K-ATPase is the known cellular target for Ouabain, several evidences suggest that its actions are not fully correlated with Na,K-ATPase ion flow inhibition, as some authors have shown that the activation of signaling pathways by Ouabain occurred even under normal Na,K-ATPase transporting activity [23–26].

Evaluating human monocytes in culture, it has been described that treatment with 100 nM Ouabain induces a broad modulatory effect on these cells. Ouabain induced CD14 downregulation [10, 11] and prevented the size increase resulting from adhesion-induced activation and the appearance of the proinflammatory CD14^+^/CD16^+^ subpopulation [11]. At the same time, monocytes exposed to Ouabain upregulated the expression of several molecules associated with the activation process such as CD69, CD80, CD86, and HLA [11, 15]. Increased cytokine levels of IL-1 beta, TNF-*α* [13–15], and IL-10 [15] were observed in monocyte cultures exposed to Ouabain. Various signaling pathways have been ascribed for the different effects observed [10, 11, 13].

Nevertheless, despite so many actions described for Ouabain in different cells of the immune system, there is no, to our knowledge, published data concerning possible effects of this substance on dendritic cells. Thus, owing to the great importance of this cell type on the control of proper immune responses, it was of our interest to analyze whether Ouabain could influence the maturation and functional ability of dendritic cells.

## 2. Material and Methods

### 2.1. DC Differentiation

The ethics committee of the Hospital Universitário Clementino Fraga Filho-UFRJ agreed with the study protocol, which is registered under the approval number 148/09. Blood samples were obtained from peripheral blood samples collected from healthy volunteers using sodium heparin (Roche, Rio de Janeiro, Brazil) as anticoagulant. Peripheral blood mononuclear cells (PBMC) were isolated by density gradient centrifugation with Ficoll-Paque (GE, USA). PBMC were seeded at a concentration of 5 × 10^6^ cells per well (1 mL final volume) for 2 hours in 24-well plates (TPP, Switzerland), in humidified chamber with 5% CO_2_ atmosphere at 37°C. Afterwards, nonadherent cells were removed by extensive washing. Adherent cells were cultured in RPMI1640 medium (Sigma, Chemical Co., USA) supplemented with 10% fetal bovine serum (FBS, 500 *μ*L final volume) with or without 50 ng/mL GM-CSF (PeproTech, USA) and 50 ng/mL IL-4 (R&D Biosystems, USA), for 5 days. In some experiments, cells were preincubated with 100 nM Ouabain (Sigma, Chemical Co., USA) for 24 h, followed by the addition of fresh medium or the cytokines described above.

### 2.2. DC Activation

Dendritic cells, obtained after 5 days of culture, had their culture medium replaced by fresh medium containing GM-CSF and IL-4 in the presence or absence of 50 ng/mL TNF-*α* (R&D Biosystems, USA), for further 48 h. In some experiments Ouabain (10 pM, 1 and 100 nM) or the p38 inhibitor SB202109 (20 *μ*M, Sigma Chemical Co., USA) was added during the activation period (48 hours).

### 2.3. DC Phenotypic Analysis

To determine CD14, CD1a, CD83, CD86, CD80, or HLA-DR expression, both after DC differentiation and activation, cells were collected and incubated for 10 min with phosphate buffered saline (PBS) + FBS 5% solution. Cells were stained for 30 min at 4°C with FITC conjugated anti-CD14, FITC conjugated anti-HLA-DR, PE conjugated anti-CD1a, PE conjugated anti-CD86, PE conjugated anti-CD80, and PE conjugated anti-CD83 (all from BD Biosciences, USA). After the incubation period, cells were washed with PBS and analyzed by flow cytometry (FACSCalibur, Becton and Dickinson, USA) and data analyses were performed via the software Summit 4.3 (Dako, USA).

### 2.4. Cytokine Production Evaluation

DCs were differentiated for 5 days, and then cells were incubated with GM-CSF, IL-4, and TNF-*α* or LPS (100 ng/mL) in the presence or absence of Ouabain (100 nM) for further 48 h. Supernatants were collected and stored at −20°C until use. IL-12p40 and IL-10 concentrations were measured with enzyme-linked immunosorbent assay (Duo-Set kits, purchased from R&D Systems, USA), according to the manufacturer's instructions. Optic density was read at 450 nm in a microplate reader Sunrise Basic (Tecan, Austria).

### 2.5. Cell Viability Assay

Despite the fact that the MTT assay depends on the metabolic activity of the cell due to NAD(P)H flux it may be used for cell viability determination, as mitochondrial activity is usually related to the number of viable cells. In the present experiments, 2.5 × 10^6^ cells were plated per well in 96-well plates (TPP, Switzerland) for 5 days. After DC differentiation, cells had their culture medium replaced by fresh medium containing GM-CSF, IL-4, and TNF-*α* in the presence or absence of Ouabain (10 pM, 1 and 100 nM) for 48 h. Then, cells were incubated with 0.5 *μ*g/mL MTT (Sigma Chemical Co., USA) for 4 h. After that, the supernatant was removed and formazan crystals dissolved with 200 *μ*L of dimethyl sulfoxide (DMSO). Optical density was read at 490 nm in a microplate reader Sunrise Basic (Tecan, Austria).

### 2.6. Western Blot

After DC differentiation cells were incubated with GM-CSF, IL-4, and TNF-*α* in the presence or absence of Ouabain (100 nM) for 10 min. The activation of NF*κ*B was determined by Western blot analysis. After determination of protein concentration by the Bradford method [27], samples (60 *μ*g/lane) were subjected to sodium dodecyl sulfate-polyacrylamide gel electrophoresis (9%) and transferred to polyvinylidene difluoride (PVDF) membranes. The membranes were incubated overnight with rabbit anti-phospho-NF*κ*B antibody (1 : 1000) or mouse anti-actin (1 : 5000). Both antibodies were obtained from Santa Cruz Biotechnology (CA, USA). The membranes were washed in TBS and were then exposed to horseradish peroxidase-conjugated secondary anti-rabbit or anti-mouse IgG antibody (1 : 2000, Bio-Rad Laboratories Inc., CA, USA) at room temperature for 60 minutes. Detection was performed on L-Pix Chemi photodocumentation system (Loccus, SP, Brazil) using Luminata Millipore (MA, USA). The density of protein bands was analyzed by densitometry with ImageJ. The mean value for the control was set at 100%.

### 2.7. Endocytosis Assay

Phagocytic capacity was assessed by FITC-dextran uptake. After DC differentiation cells were incubated with GM-CSF, IL-4, and TNF-*α* in the presence or absence of Ouabain (100 nM) for further 48 h. Then, cells had their culture medium replaced by fresh medium containing FITC-dextran (40 kDa, 0.5 mg/mL, Sigma Chemical Co., USA). Half of the cells were incubated at 4°C to assess the background uptake in order to allow the determination of the background staining. Following 1 h incubation at 37°C, cells were washed twice in ice-cold PBS and subjected to flow cytometric analysis.

### 2.8. Mixed Lymphocyte Reaction

After 48 h of activation with 50 ng/mL TNF-*α*, in the presence or absence of 100 nM Ouabain, DCs were tested for allostimulatory ability. For that, 10^5^ lymphocytes were cultured in 96-well microplates (round-bottomed) with different concentrations of allogeneic DCs (1 : 4, 1 : 40, and 1 : 400) at 37°C in a 5% CO_2_ atmosphere. Thymidine incorporation was measured on day 5 by 6 h pulse with ^3^H-thymidine (1 *μ*Ci/well, Amersham Life Sciences) and then harvested using a multiwell cell harvester. ^3^H-thymidine incorporation was evaluated by liquid scintillation counting, using a TriCarb 1600CA counter (Packard, Inc., USA).

### 2.9. Plasma Membrane Potential Analysis

To investigate whether Ouabain could modulate plasma membrane potential during DC activation, dendritic cells were incubated with an anionic lipophilic potential-sensitive dye, namely, bis-oxonol (bis-[1,3-dibutylbarbituric acid] trimethineoxonol) or DiBAC_4_(3), obtained from Molecular Probes (USA) by the method described elsewhere [28]. Briefly, differentiated DCs were incubated for 30 minutes with 250 nM DiBAC_4_(3), at 37°C with 5% CO_2_ atmosphere, in the absence or presence of 100 nM or 1 mM Ouabain. Immediately after this period, cells were examined by flow cytometry in a FACSCalibur equipment (Becton and Dickinson, USA). Alternatively, immature DCs were cultured with or without TNF, in the absence or the presence of 100 nM Ouabain, for 48 h and further incubated with 250 nM DiBAC_4_(3) at 37°C with 5% CO_2_ atmosphere. Then, the experiment proceeded as described above. As a positive control for membrane depolarization, DCs were incubated for 30 minutes with 50 mM KCl. The excitation was performed with a 488 nm argon laser and fluorescent emission was detected at 530 nm. Ten thousand cells were examined under each condition, and data analyses were performed via the software Summit 4.3 (Dako, USA).

### 2.10. Evaluation of Na,K-ATPase Activity

Ouabain is a known inhibitor of Na,K-ATPase. To verify if, in our model, Ouabain could inhibit the enzyme, immature DCs were cultured for 48 h in the presence or absence of 100 nM Ouabain. Additionally, part of the cells received 100 nM Ouabain only during membrane preparation. The ATPase activity was measured as follows.

[^32^P]P_i_ was obtained from the Brazilian Atomic Energy Institute (IPEN). The enzymes used in the synthesis of [*γ*-^32^P]ATP (glyceraldehyde-3-phosphate dehydrogenase, glycerol-3-phosphate dehydrogenase, triose-phosphate-isomerase, 3-phosphoglycerate kinase, and lactate dehydrogenase) were from Boehringer Mannheim (Darmstadt, Germany). The radioactive ATP was prepared according to Walseth and Johnson [29] with modifications described by Maia et al. [30]. Protein measurements were performed using 1 mg/mL BSA as a standard.

Microsomal fractions from Ouabain-treated DCs were obtained through differential centrifugation with a Beckman Ultracentrifuge. Briefly, all cells (approximately 1.4 × 10^6^) were submitted to hypotonic lysis in ET buffer (Tris HCl 10 mM + 2 mM EDTA). The material was spun down at 2500 rpm/5 min to avoid nuclei and debris and spun down again with 8000 rpm/20 minutes to avoid mitochondria. The membrane fraction containing Na,K-ATPase activity was obtained through collection and homogenization of the pellet from a final centrifugation with 50000 rpm/90 minutes. The pellet was resuspended in ISE buffer (12.5 mM imidazole + 250 mM sucrose + 2 mM EDTA) and stored at −22°C to be used in activity determinations. The Na,K-ATPase activity was assayed by measuring the release of ^32^Pi from [*γ*-^32^P]ATP hydrolyzed by the microsomes isolated from DCs as described before [31, 32]. Briefly, 45 *μ*g aliquots of the microsomal preparation were diluted to a final volume of 0.5 mL in 10 mM Tris-HCl, pH 7.0, 130 mM NaCl, 20 mM KCl, 5 mM MgCl_2_, and 02 mM EGTA. The reaction was initiated by the addition of 3 mM ATP/[*γ*-^32^P]ATP (specific activity of 1520 cpm/nmol) and allowed to proceed for 40 min at 37°C. The reaction was stopped by the addition of 0.2 mL 0.4 M perchloric acid and the tubes were placed in an ice bath. After the addition of 0.2 mL of ice-cold water and 400 *μ*L of activated charcoal, the tubes were centrifuged at 2000 rpm for 10 min, and 0.5 mL of supernatant was then collected with a Pasteur pipette and spotted on a filter paper disk. The ^32^Pi released was quantified by liquid scintillation counting in a Packard Tri-Carb 2100 LSC scintillation counter. All measurements described were performed without and with 1 mM Ouabain, the difference in activity corresponding to Na,K-ATPase activity. Our DCs membrane preparation exhibits an ATPase activity of 300 to 339 nmol/mg/min.

### 2.11. Statistical Analysis

Statistical analysis was performed using one way ANOVA and Student's *t*-test. Values of *P* ≤ 0.05 were considered statistically significant.

## 3. Results

### 3.1. Effect of Ouabain on DC Differentiation

Our group has previously reported the finding that human monocytes exhibited CD14 downregulation after incubation with 100 nM Ouabain for 24 h [10, 11, 15]. Such finding was not related to induction of cell death but seemed to be part of a broad modulatory regulation mainly of undifferentiated monocytes [11]. Nevertheless, a classical situation where monocytes may lose CD14 expression is the differentiation into dendritic cells. During differentiation, in parallel to decreasing CD14, monocytes progressively begin to express the surface molecule CD1a, a process that can be reproduced* in vitro*, lasts around 5 days, and leads to the differentiation of CD11c^+^CD11b^+^ myeloid DCs [6, 33, 34]. Thus, to investigate the possibility that monocytes pretreated with Ouabain are more prone to dendritic cell differentiation, these monocytes were further exposed to IL-4 and GM-CSF, a standard protocol for DC differentiation* in vitro* [33].

**Figure 1:**
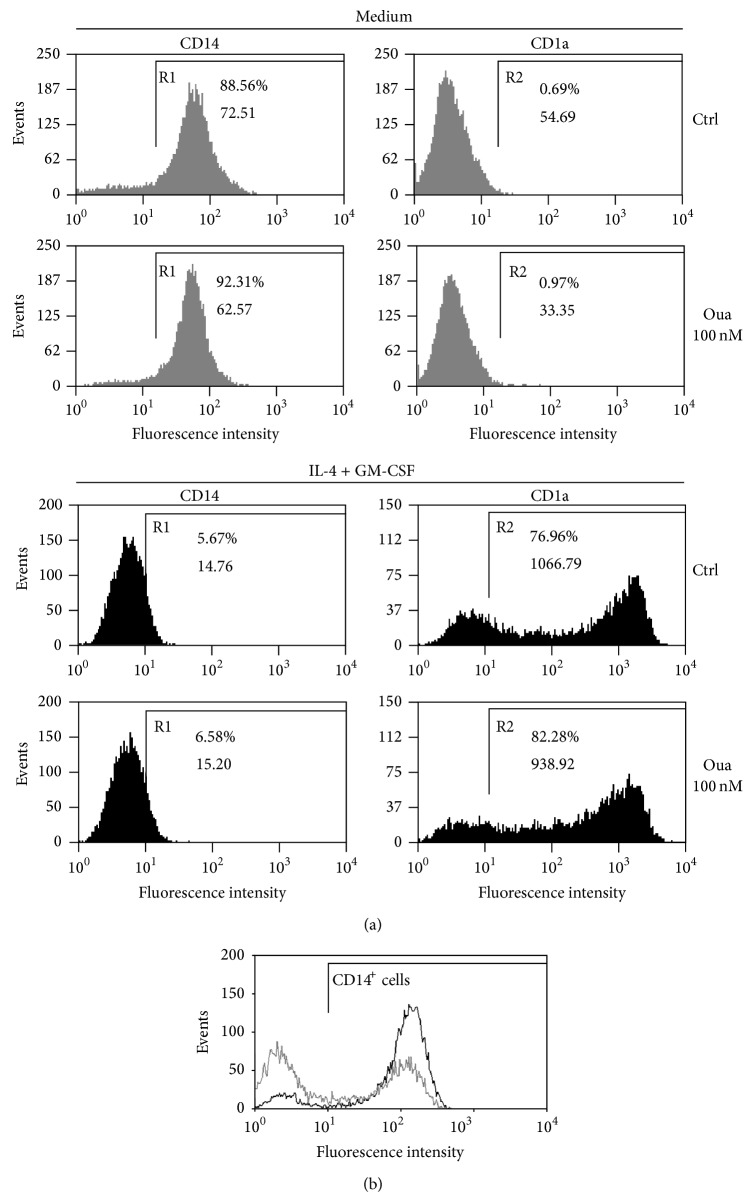
CD14 and CD1a expression patterns on monocytes cultured with Ouabain (Oua) prior to differentiation into dendritic cells. (a) Cells were incubated with medium in the presence or absence of 100 nM Oua for 24 h and then with medium or IL-4 + GM-CSF (50 ng/mL) for 5 days to allow monocyte differentiation into DC. Following this period, CD14 and CD1a expression was analyzed by flow cytometry. Representative histograms of 4 independent experiments showing CD14 and CD1a expression patterns in cells incubated only with medium, IL-4 + GM-CSF, and in cells treated previously with 100 nM Oua. R1 and R2 regions comprise positive cells for CD14 and CD1a, respectively. The percentage of positive cells and mean fluorescence intensity is indicated in each histogram. (b) Monocytes were incubated for 24 h with 100 nM Oua. Representative experiment showing CD14 expression patterns in control cells (black histogram) and in cells treated with 100 nM Ouabain for 24 h (gray histogram).

In the present work monocytes were cultured in the presence or absence of 100 nM Ouabain, for 24 h. Following this exposure to Ouabain, cells were washed and cultured with fresh medium for a further 5 days. As depicted in Figure 1(a) (top panel, gray histograms), it was observed that after this period in the absence of Ouabain, monocytes restored CD14 protein to control levels (control, 88.56%; Oua, 92.31%), when comparing to the values obtained just after the 24 h exposure to Ouabain. In this case, the percentage of positive cells following Ouabain treatment was 49.42% versus 87.22%, in control (Figure 1(b)). Furthermore, as seen in Figure 1(a), no CD1a expression was observed after 5 days, suggesting that Ouabain per se did not induce the process of differentiation.

Additionally, Figure 1(a) (bottom panel, black histograms) also indicates that when monocytes were stimulated to differentiate into dendritic cells with IL-4 and GM-CSF, there was, as expected, an intense decrease of the percentage of CD14^+^ cells, as well as an increase in the expression of CD1a. However, the same values were achieved when cells differentiate following Ouabain pretreatment. Thus, it seems that Ouabain-induced CD14 downregulation does not reflect facilitation towards DC differentiation.

### 3.2. Modulation of DC Surface Phenotype by Ouabain during Activation

Several authors have reported that Ouabain induces the activation of signaling pathways [13, 24, 35], for instance, p42/44 MAPK and p38 MAPK. Our group described that Ouabain may induce or inhibit the activation of p38 MAPK, in a cell type dependent manner [10, 12]. Given that p38 MAPK function is essential for dendritic cells activation [36], it is possible to hypothesize that Ouabain could modulate another stage of DCs, namely, its final maturation or activation via p38 signalling.

The expression of a number of surface molecules was studied in the presence or absence of Ouabain to verify any influence on the activation process.

**Figure 2:**
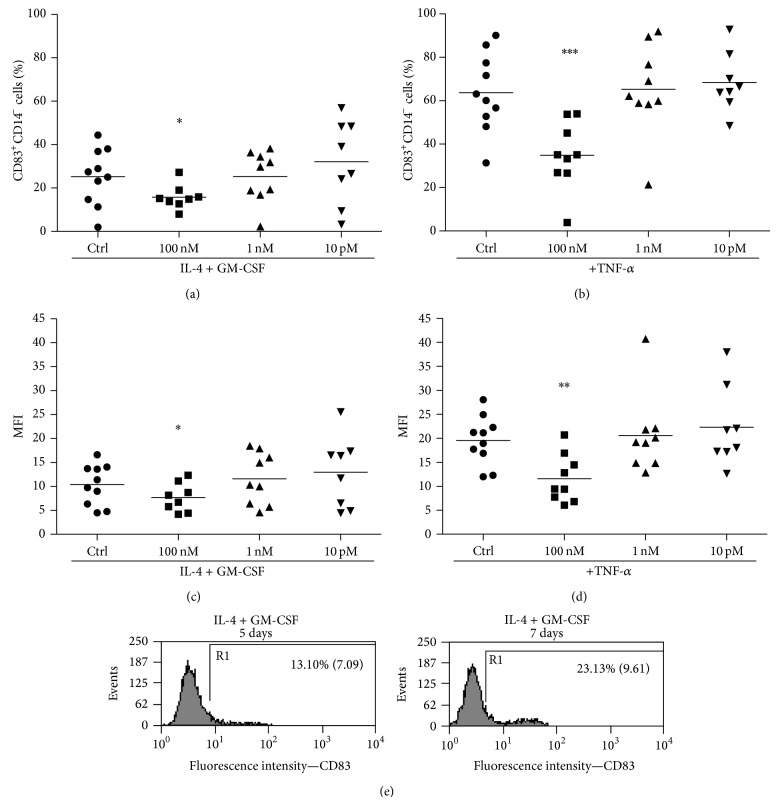
Ouabain effect on CD83 expression in dendritic cells during activation. After the differentiation period (5 days), cells were incubated for further 48 h in the presence (b, d) or absence of TNF-*α* (50 ng/mL) (a, c) and in the presence or absence of 100 nM, 1 nM, or 10 pM Oua. CD83 expression was analyzed by flow cytometry. Data are expressed as the percentage of CD14^−^ CD83^+^ cells ((a) and (b) panels) or the means of fluorescence intensities (MFI: (c) and (d) panels), and lines denote the means of at least eight independent experiments. (e) Cells were incubated for 5 or 7 days with IL-4 + GM-CSF, and CD83 expression was analyzed. The percentage of positively stained cells (R1) and their means of fluorescence intensities are indicated in the representative histogram. ∗, ∗∗, and ∗∗∗ are significantly different from the control (^∗^ *P* < 0.05; ^∗∗^ *P* < 0.01; ^∗∗∗^ *P* < 0.001).

First of all, we tested the viability of immature DCs (after differentiation) treated in the presence of Ouabain and TNF-*α* for 48 h, but no difference was found in any of the concentrations used in our experiments (Table 1). The following step was to analyze whether Ouabain could influence the activation process of DCs. Mature DCs are typically characterized by the expression of a number of surface molecules that may facilitate the interaction and activation of T cells. However, despite the fact that the function of CD83 molecule is not clear, its expression after an activation stimulus is associated with many aspects of DCs activation. Thus, in order to investigate whether Ouabain could influence this process, immature DCs were stimulated with TNF-*α* in the absence and in the presence of pharmacological (100 nM) or physiological (1 nM and 10 pM) concentrations of Ouabain, and the expression of CD83 was analyzed (Figure 2). To assure that only differentiated DCs would be evaluated, we employed a gate in the CD14 negative population. As it can be observed, the presence of 100 nM Ouabain for 48 h induces a significant decrease in CD83 expression independently of TNF-*α* stimulus (Figures 2(a) and 2(c)). This is observed both in terms of percentage of positive cells as well as fluorescence intensity. In the presence of TNF-*α*, 100 nM Ouabain was able to inhibit CD83 expression, but no effect was observed when using physiological concentrations of this hormone (Figures 2(b) and 2(d)). As shown in Figure 2(e), our protocol promoted the generation of relatively immature, nonactivated DC, with very low expression of CD83. When we compared CD83 expression of immature DCs on day 5 and on day 7 of culture, we observed an increase in this expression, suggesting that culture conditions per se induce some expression of this molecule. Thus, these data indicate that Ouabain interferes with CD83 upregulation induced by either TNF-*α* or that promoted by prolonged incubation* in vitro* (Figure 2(e)).

The concept of DC maturation/activation relies on MHC upregulation. The activation stimuli modify both the turnover and the gene expression of MHC, consequently altering the number of MHC molecules expressed at the cell membrane [33, 37].

**Figure 3:**
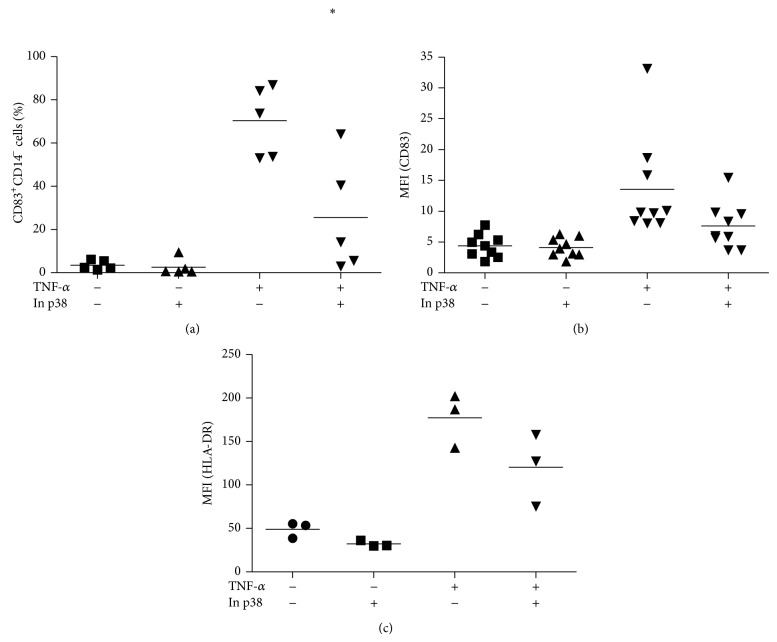
CD83 and HLA-DR expression on dendritic cells during activation. After the differentiation period (5 days), cells were incubated for further 48 h with or without TNF-*α* (50 ng/mL), in the presence or absence of 20 *μ*M of p38 inhibitor (SB202109). CD83 and HLA-DR expression were analyzed by flow cytometry. Data are expressed as (a) the percentage of CD83^+^ cells, or (b) MFI (mean of fluorescence intensity) of CD83^+^ cells, (c) MFI of HLA-DR^+^ cells, and lines denote the means of 3–5 independent experiments. ∗ is significantly different from TNF-*α* (^∗^ *P* < 0.05).

As mentioned above, p38 phosphorylation/activation is essential for activation of DCs. Aspects such as the expression of surface molecules and cytokine production are severely affected when p38 MAPK is inhibited [36]. Here, we evaluated the expression of CD83 and HLA-DR under p38 inhibition. As expected, the inhibitor of p38 was able to inhibit both CD83 and HLA-DR expression in the presence of TNF-*α* (Figures 3(a), 3(b), and 3(c), resp.).

**Figure 4:**
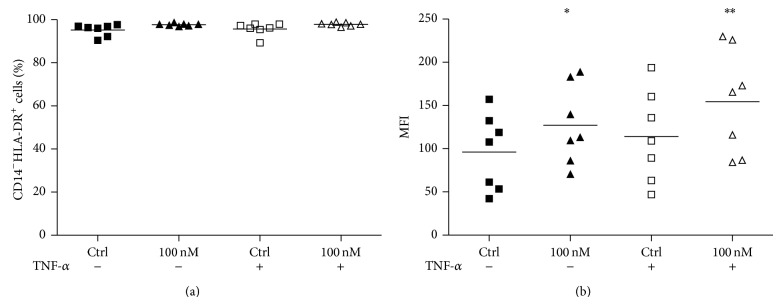
HLA-DR expression on dendritic cells during activation. After the differentiation period (5 days), cells were incubated for further 48 h with or without TNF-*α* (50 ng/mL), in the presence or absence of 100 nM Oua. HLA-DR expression was analyzed by flow cytometry. Data are expressed as (a) the percentage of HLA^+^ cells, or (b) MFI (mean of fluorescence intensity), and lines denote the means of seven independent experiments. ∗ and ∗∗ are significantly different from the control (^∗^ *P* < 0.05; ^∗∗^ *P* < 0.01).

We then explored the possibility that Ouabain could produce impairment of the activation process and, therefore, affect the surface expression of HLA molecules. For that, we employed the same protocol as in Figure 2, but we evaluated HLA-DR expression under 100 nM Ouabain influence. As it can be observed, virtually all immature DCs express HLA-DR (Figure 4(a)). Moreover, cell surface levels of HLA-DR increased under exposure to TNF-*α* (as depicted by the fluorescence intensity of positive cells) and were even higher in cells treated with TNF-*α* plus Ouabain (Figure 4(b)). We also observed that Ouabain alone was capable of inducing a significant increase of HLA-DR levels (Figure 4(b)).

**Figure 5:**
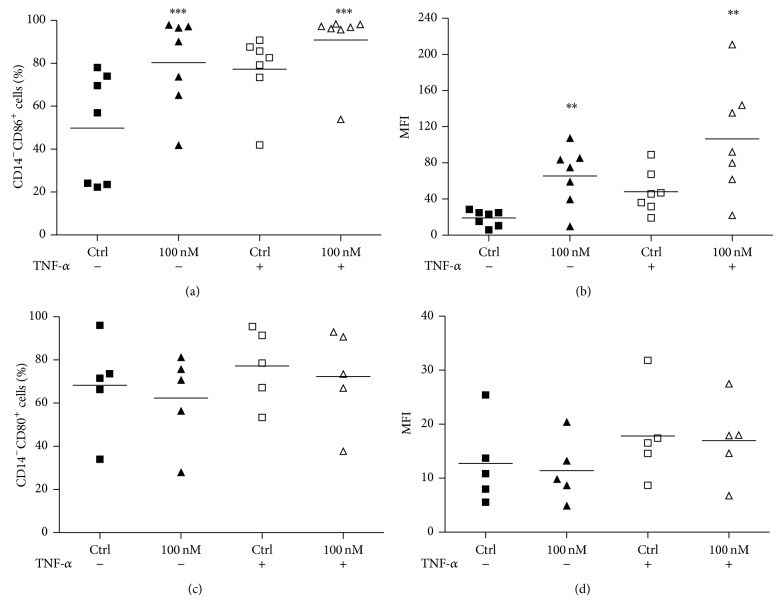
CD86 and CD80 expression on dendritic cells during activation. After the differentiation period (5 days), cells were incubated for further 48 h with or without TNF-*α* (50 ng/mL), in the presence or absence of 100 nM Oua. CD86 and CD80 expression was analyzed by flow cytometry. Data are expressed as (a, c) the percentage of positive cells, or (b, d) MFI (mean fluorescence intensity), and lines denote the means of five independent experiments. ∗∗ and ∗∗∗ are significantly different from the respective controls (^∗∗^ *P* < 0.01; ^∗∗∗^ *P* < 0.001).

Besides HLA-DR, the expression of costimulatory molecules also defines the state of activation of DCs. The balance of these costimulatory molecules has been pointed out as important for differentiating different T helper lymphocytes and seems to be dictated by microenvironmental conditions [38]. Similar to that observed for HLA-DR, there was an increase in the expression of CD86 per cell, measured as fluorescence intensity of this protein (Figure 5(b)). In addition, an increase in the percentage of CD86^+^ cells was also observed (Figure 5(a)). As it can be perceived, both incubations with Ouabain alone and along with TNF-*α* induce the upregulation of CD86. However, contrary to that seen for CD86, the expression of CD80 under these conditions remained unchanged (Figures 5(c) and 5(d)).

### 3.3. Ouabain Influence in Cytokine Production during DC Activation

In addition to differences in surface receptors, a key feature observed in mature DCs is the production of IL-12, a critical cytokine for the subsequent Th1 polarization of naive T cells encountered in secondary lymphoid tissues and whose lack of production drives the differentiation of Th2 lymphocytes [39]. So, considering the importance of IL-12 in the continuity of the immune response, we then looked whether Ouabain might impact its secretion by DCs.

**Figure 6:**
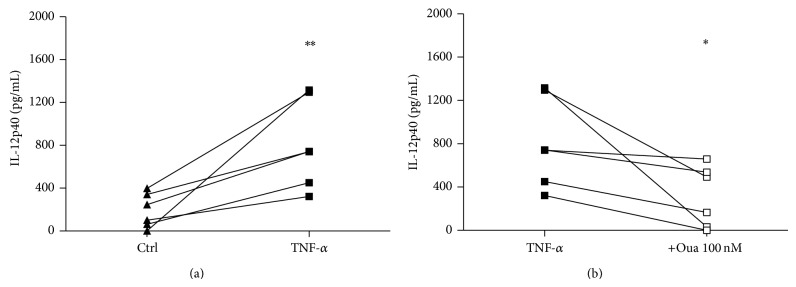
IL-12 secretion during DC activation. After the differentiation period (5 days), cells were incubated for further 48 h with or without TNF-*α* (a) (50 ng/mL) or with TNF-*α* in the presence or absence of 100 nM Oua (b). Then, the supernatants were collected and assayed for IL-12p40 production by ELISA. Data are expressed as cytokine concentrations (pg/mL) from six distinct individuals. ∗ and ∗∗ are significantly different from the respective controls ((a): without TNF, (b): without Oua) (^∗^ *P* < 0.05; ^∗∗^ *P* < 0.01).

In order to address this matter, supernatants were collected from differentiated dendritic cells (maintained 5 days in the presence of IL-4 and GM-CSF) after further 48 h activation with TNF-*α* or TNF-*α* plus Ouabain, and then IL-12 secretion was measured. As depicted in Figure 6, significant amounts of IL-12 were secreted by DCs activated with TNF-*α* for 48 h. However, DCs stimulated with TNF-*α* along with 100 nM Ouabain produced lower levels of IL-12, suggesting that Ouabain significantly inhibits the secretion of this cytokine during TNF-induced DC maturation.

Impaired dendritic cell IL-12 production can be accompanied by IL-10 upregulation, when cells had their phenotype directed toward a regulatory pattern [40]. Therefore, IL-10 secretion was measured under the same conditions, and LPS stimulus was employed as a positive control for IL-10 production. We found, as expected, that LPS (used as a positive control) induced IL-10 secretion after 7 days (>1000 pg/mL), whereas TNF-*α* had no effect. Similarly, Ouabain (100 nM) alone was not able to induce IL-10 production by dendritic cells nor to modify TNF-*α* effect (data not shown).

### 3.4. Activation of NF*κ*B

**Figure 7:**
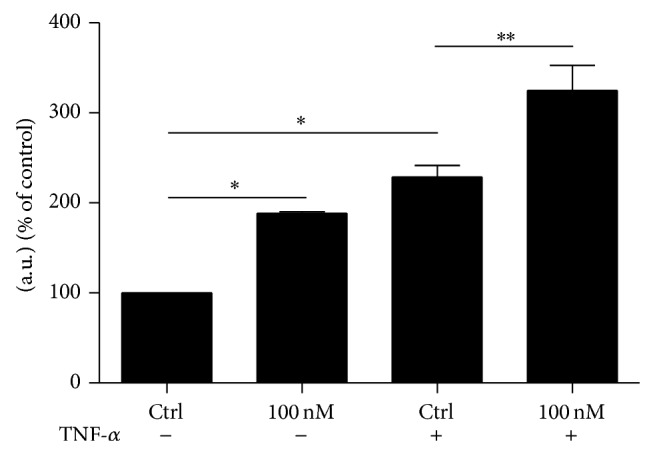
NF*κ*B activation during DC activation. After the differentiation period (5 days), cells were incubated for 10 min with or without TNF-*α* (50 ng/mL), in the presence or absence of 100 nM Oua. Then, the phosphorylation of NF*κ*B was evaluated by Western blot. Signals are expressed in arbitrary units (% of control) as the mean ± SEM of 3 independent experiments (^∗^ *P* < 0.05; ^∗∗^ *P* < 0.01).

The results obtained to this point demonstrate that incubation with Ouabain during DCs activation only partially reproduces the phenotype observed for p38 inhibitor treatment. To test the hypothesis that Ouabain modulates other important signaling pathways related to HLA-DR and CD86 upregulation, we evaluated the activation of the transcription factor NF-*κ*B. We can observe in Figure 7 that TNF-*α* stimulus, as expected, induced an increase in NF-*κ*B phosphorylation. Besides, treatment of DCs with pharmacological concentration of Ouabain (100 nM) induced an increase in NF-*κ*B phosphorylation, both in the presence or absence of TNF-*α* (Figure 7).

### 3.5. Analysis of Plasma Membrane Depolarization

**Figure 8:**
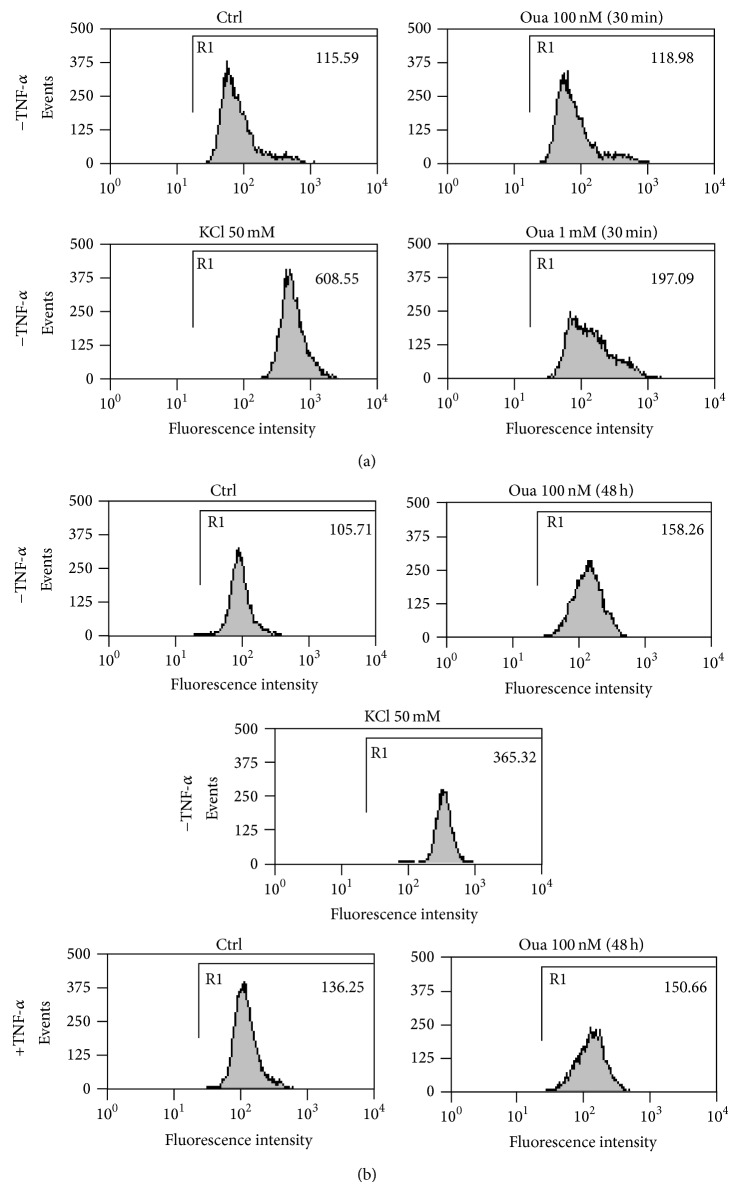
Evaluation of plasma membrane potential in the presence of Ouabain. (a) Differentiated DCs (maintained 5 days with GM-CSF and IL-4) were further incubated in the presence or absence of 100 nM Ouabain or 1 mM Ouabain with 250 nM of the fluorescent oxonol dye DIBAC_4_(3) for 30 min. (b) Differentiated DCs were incubated with or without TNF-*α* in the presence or absence of 100 nM Ouabain for further 48 h. Then, cells were washed, free medium was added, and DCs were incubated with 250 nM DIBAC_4_(3) for 30 min. As a control of membrane depolarization, 50 mM KCl was added for 30 min (during incubation with DIBAC_4_(3)) to the cultures. Next, cells were analysed using flow cytometry, as indicated in Material and Methods section. Data depicts a representative experiment from one individual ((a), 5 independent experiments; (b), 2 independent experiments) and values in each panel refer to the means of fluorescence intensities (MFIs) of DIBAC_4_(3).

Inhibition of Na,K-ATPase may lead to plasma membrane depolarization and ultimately to a collapse of the Na^+^ and K^+^ gradients across the cell membrane of animal cells [41]. Although prolonged impairment of Na,K-ATPase is related to cell death, mainly induced by lack of K^+^ [28, 42], several authors have described Ouabain effects unrelated to the modulation of ion fluxes, induced via the activation of cell signaling pathways [23–26]. So, it was important to know whether in our experimental model the concentration of 100 nM Ouabain could promote plasma membrane depolarization, an indicative of a significant inhibition of the Na,K-ATPase enzyme, during DC activation. For that, we made use of the fluorescent probe DIBAC_4_(3), a bis-oxonol dye that enters in cells according to their membrane polarization and incubated distinct subsets of DCs. The first group of experiments tested an immature group of DCs without TNF-*α*. The experiments were performed incubating 100 nM or 1 mM Ouabain for 30 minutes. As depicted in the representative experiment (Figure 8(a)) only the positive control KCl (50 mM) and 1 mM Ouabain induced significant plasma membrane depolarization. However, when DCs were incubated with 100 nM Ouabain for 48 h, with or without TNF-*α*, a partial depolarization could be observed even with this concentration of Ouabain (Figure 8(b)).

### 3.6. Inhibition of Na,K-ATPase Activity

Ouabain is a classical inhibitor of Na,K-ATPase activity. When the ATPase activity was measured using a preparation of microsomes isolated from dendritic cells exposed for 48 h to 100 nM Ouabain, it was possible to obtain an inhibition of approximately 50% of the activity (Table 2).

### 3.7. Evaluation of DC Function under Ouabain Treatment

As a result of the modifications observed in DCs in the presence of Ouabain, we evaluated fundamental functions of immature and mature DCs. Antigen capture using dextran was chosen to look for phagocytosis of immature DCs and the stimulatory capacity of DCs to elicit proliferative responses of alloreactive T lymphocytes was used as a function of mature DCs.

**Figure 9:**
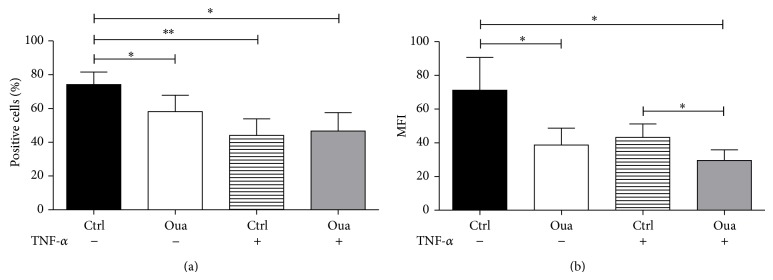
Phagocytic activity of dendritic cells cultured with Ouabain. After the differentiation period (5 days), cells were incubated for further 48 h with or without TNF-*α* (50 ng/mL), in the presence or absence of 100 nM Oua. After this period, cells were incubated with FITC-dextran (0.5 mg/mL) for 1 h at 37°C and analyzed by flow cytometry. Data are expressed as the percentage of positive cells and MFI (mean fluorescence intensity) of positive cells (mean ± SEM of 6 independent experiments). ∗ and ∗∗ are significantly different from respective controls ((a): without TNF; (b): without Oua) (^∗^ *P* < 0.05; ^∗∗^ *P* < 0.01).

FITC-dextran uptake after 7 days was measured. As depicted in Figure 9, around 75% of immature DCs captured FITC-dextran particles (Figure 9(a)). Strikingly, in the presence of 100 nM Ouabain (added in the last 48 h of culture) the percentage of DCs able to capture FITC-dextran and the phagocytic capacity of these cells decreased nearly 20% and 45%, respectively.

As a consequence of activation, dendritic cells downregulate their machinery of antigen capture. As shown in Figures 9(a) and 9(b), TNF-*α* stimuli (present for the last 48 h of culture) diminished FITC-dextran uptake by dendritic cells, as seen by the 40% decrease in the percentage of positive cells and also in the MFI. In the presence of Ouabain, the downregulation promoted by TNF-*α* also occurred, supporting the notion that Ouabain does not inhibit DC activation in many aspects. Furthermore, the coincubation of Ouabain with TNF-*α* promoted an additional effect on DC ability of capturing dextran particles, demonstrated by 33% decrease in MFI levels, when compared to that observed with TNF-*α* alone (Figure 9(b)).

**Figure 10:**
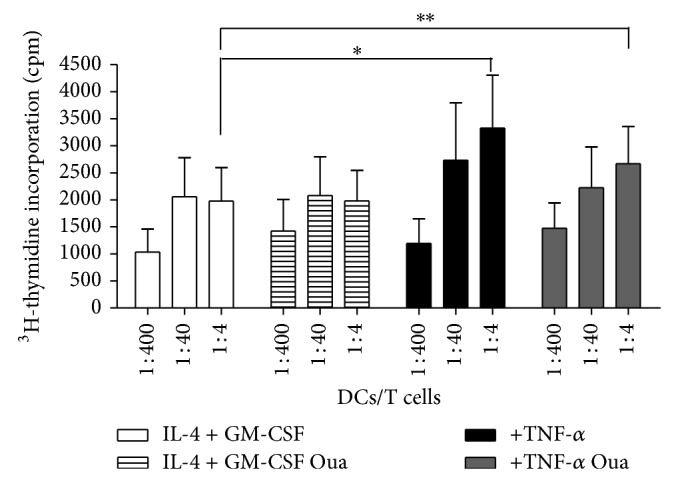
Evaluation of mixed lymphocyte reaction of immature and mature dendritic cells previously incubated with Ouabain. DCs treated with or without 100 nM Oua were cocultured with allogeneic lymphocytes (10^5^ cells/well) at indicated cell ratio, for 5 days. Proliferation was assessed in terms of H^3^-thymidine incorporation, added for 6 h after the culture period. Data in bars are expressed as mean incorporation ± SEM of five independent experiments in triplicate (^∗^ *P* < 0.05; ^∗∗^ *P* < 0.01).

In Figure 10 is depicted the means of H^3^-thymidine incorporation after five days of coincubation of DCs with lymphocytes. DCs induced allogeneic T-cell proliferation and, as expected, stimulation with TNF-*α* led to higher levels proliferation at the cell ratio 1 : 4 (DCs/T cells) compared to unstimulated/immature DCs. The exposure of immature DCs to Ouabain for 48 h did not modify the stimulatory capacity of DCs. Moreover, the stimulatory ability of Ouabain-treated mature DCs was superior to that of control immature DCs, indicating that DCs had this capacity preserved. These data support the notion that despite the reduction of IL-12 secretion and CD83 expression Ouabain treatment does not impede the T cell stimulatory capacity of DCs.

## 4. Discussion

In the present work, it was observed that Ouabain (100 nM)* per se* did not induce the process of differentiation of monocytes into DCs. Corroborating previous results [10] the decrease in CD14 levels, seen after 24 h incubation with Ouabain, was reversed and went back to control values after Ouabain removal and the maintenance of monocytes in culture in fresh medium for further 5 days. Additionally, no CD1a expression was observed under these conditions. Although the incubation with 100 nM Ouabain during the whole process of differentiation induced high toxicity in DCs (data not shown), pretreatment with Ouabain did not impair monocyte differentiation into immature DCs, using IL-4 + GM-CSF for 5 days. Altogether, the present data clearly demonstrates that Oua-induced CD14 downregulation in human monocytes is completely unrelated to differentiation into DCs.

Decreased surface levels of CD14 may result from an increased cleavage of mCD14 into sCD14 and imply monocyte activation [43, 44]. Similarly, monocytes exposed to Ouabain expressed the surface molecules CD69, CD80, CD86, and HLA [11, 15] also related to the activation process, as well as increased cytokine levels [13–15]. However, these monocytes did not induce an increased allogeneic activation in a mixed leukocyte response or an increase in the percentage of phagocytic cells [15].

Immature DCs may be activated by TNF-*α* and the possibility existed that this process could be modulated by Ouabain (100 nM). Activated/mature DCs decrease their phagocytic activity and in the presence of Ouabain the downregulation promoted by TNF-*α* also occurred. Similarly, TNF-*α* led to an increased ability to induce allogeneic T-cell proliferation and the simultaneous incubation with Ouabain did not affect the stimulatory capacity. Therefore, in functional terms the activation/maturation of DCs in the presence of Ouabain was not modified. This was not so when the expression of molecules intrinsically related to DC activation, such as CD83, HLA, and CD86 was analyzed. Various patterns emerged when immature DCs were activated with TNF-*α* in the presence or absence of Ouabain. The increase in CD83 expression was inhibited, whereas HLA and CD86 were significantly upregulated by Ouabain. Another feature of DC activation is the secretion of IL-12 and Ouabain inhibited the secretion of this cytokine.

Taken together, these results clearly demonstrate that Ouabain modulates some of the changes observed during the maturation/activation process of DCs by TNF-*α*; it also suggests that markers normally ascribed to activated cells are independently regulated and do not equally impact on some functional aspects. For example, DCs stimulated with TNF-*α* or TNF-*α* plus Ouabain induced similar levels of lymphocyte proliferation, regardless whether the cells expressed CD83. Thus, it appears that CD83 upregulation during DCs activation is not essential for induction of lymphocyte activation. This unexpected result is in agreement to those obtained by Chang and coworkers that reported equivalent mixed lymphocyte reaction between CD83^−^ and CD83^+^ DCs subtypes with comparable levels of CD40, CD80, CD86, and MHC class II [45]. In the present work, Ouabain treatment maintained CD80 expression while increasing CD86 expression. Costimulation via CD86 is capable of activating T cells but results in the generation of effector cells with a Th2 phenotype [38]. Therefore, Ouabain may induce Th2-driving DCs. In the present work, despite the inhibition of IL-12 production, no IL-10 was detected, raising the possibility that the presence of Ouabain may modify DC maturation towards a regulatory subtype.

A number of DCs with different phenotypes, profiles of cytokine production, and ability to stimulate different lymphocyte subpopulations have been described. Some danger signals such as ATP or TNF-*α* induce the onset of dendritic cells with a proinflammatory profile, secreting high levels of IL-12 with increased expression of CD54 and CD83 [46]. Moreover, it has been recently demonstrated that addition of the inflammatory regulator adiponectin to DCs under maturation leads these cells towards the induction of Th17 responses [47]. On the other hand, tolerogenic properties have been described for DCs matured in the presence of vitamin D(3), IL-10, TGF-*β*, or rapamycin, where DCs stimulated with these molecules present different phenotypes and patterns of cytokine production: they typically lack IL-12 production and induce low lymphocyte stimulation as well [4]. Corticoids such as dexamethasone inhibit production of IL-12 by TNF-matured dendritic cells and induce IL-10 production [4]. Moreover, dexamethasone was shown to inhibit CD86 and CD83 expression on mature DCs, without inhibiting HLA expression by these cells [4], a DC profile that also diverged from what was observed with Ouabain.

The p38 MAPK cascade is activated by TNF-*α* and LPS stimulation. It has been clearly demonstrated that the inhibition of this kinase impairs DCs activation, induced by different substances [36, 48, 49]. Häcker and coworkers reported the inhibition of CpG-induced IL-12 secretion using SB203580 (p38 MAPK-specific inhibitor). Arrighi and coworkers observed that the addition of SB203580 prevented the upregulation of CD40, CD80, CD86, HLA-DR, and CD83, under activation by LPS, TNF-*α*, and contact sensitizers [36]. In agreement, in the present work, the p38 inhibitor decreased the expression of CD83 and HLA-DR upregulated by TNF-*α*, an effect different from that produced by Ouabain. Previous reports from our group also revealed that Ouabain treatment leads to activation or inhibition of p38 MAPK in monocytes and in lymphocytes, respectively [10–12]. It has been already demonstrated that Ouabain induces several other signaling events which might induce the regulation of several genes culminating with a different pattern of modulation [24, 35]. It has been reported that the inhibition of the NF*κ*B pathway in LPS activated DCs led to the decrease of HLA-DR, CD80, CD83, and CD86 [48]. In our hands, the activation of this key signaling pathway was induced by TNF and by Ouabain, and an additive effect was observed when both substances were given together.

In our experimental model the effect of Ouabain is observed during the maturation/activation process induced in DCs by TNF-*α*. Other authors [50] described that the induction of maturation of DCs is accompanied by changes in the expression of voltage gated ion channels. By modifying the membrane potential, Ouabain may undermine the necessary adjustments to this new situation. Despite having observed a decrease in Na,K-ATPase activity, we did not observe cell death, a feature that usually correlates with sustained Na,K-ATPase impairment [28, 42].

Finally, the present work demonstrates for the first time that Ouabain is capable of modulating dendritic cell maturation and functioning at pharmacological concentrations. The results obtained so far are also relevant in terms of dendritic cell function in pathophysiological conditions where plasma Ouabain levels are usually increased, like stress, hypertension, or even pregnancy [20–22].

## Figures and Tables

**Table 1:** Viability of dendritic cells incubated with Ouabain during activation with TNF-*α*.

	Ctrl	Ouabain 100 nM	Ouabain 1 nM	Ouabain 10 pM
DCs	0.719 ± 0.118	0.729 ± 0.155	0.738 ± 0.159	0.764 ± 0.196
DCs + TNF-*α*	0.852 ± 0.184	0.797 ± 0.150	0.820 ± 0.166	0.801 ± 0.222

Cells were incubated with medium alone or with 50 ng/mL TNF-*α* for 48 h in the presence or absence of 100 nM, 1 nM, or 10 pM Oua. The viability was analyzed by MTT assay, where values refer to the means ± SD of 3 independent experiments.

**Table 2:** Na,K-ATPase activity.

	Activity (nmol/mg/min)
Ct	267.5 (±15.3)
Ouabain 100 nM (addition during membrane preparation)	71.3 (±5.7)
Ouabain 100 nM (48 h)	111.0 (±3.1)

Results are the mean (±SD) of three independent determinations. ATP hydrolysis reaction was started by the addition of 3 mM *γ* ^32^P-ATP (sp. act 1520 cpm/nmol); after 40 minutes all reactions were stopped by the addition of 0.2 mL of 0.4 M perchloric acid + 0.4 mL of activated charcoal and then centrifuged. Supernatants from these centrifugations contained ^32^Pi generated from ATP hydrolysis and specific counts were estimated through LSC in a Beckman 2100 counter. All measurements in each condition were discounted from the blanks (with acid denatured enzyme) and from the activity observed in the presence of 1 mM Ouabain to assess specific Na,K-ATPase activity. The results are the pool of cells from two donors.
